# Cyclo­hexane-1-spiro-2′-imidazolidine-5′-spiro-1′′-cyclo­hexan-4′-one

**DOI:** 10.1107/S1600536810012468

**Published:** 2010-04-14

**Authors:** T. Kavitha, S. Ponnuswamy, R. Vijayalakshmi, M. Thenmozhi, M. N. Ponnuswamy

**Affiliations:** a Centre of Advanced Study in Crystallography and Biophysics, University of Madras, Guindy Campus, Chennai 600 025, India; b Department of Chemistry, Government Arts College (Autonomous), Coimbatore 641 018, India; c Department of Chemistry, Queen Mary’s College (Autonomous), Chennai 600 004, India

## Abstract

In the title compound, C_13_H_22_N_2_O, the central imidazolidine ring is in an envelope conformation and the two cyclo­hexane rings adopt chair conformations. In the crystal structure, the mol­ecules are linked into centrosymmetric *R*_2_^2^(8) dimers by pairs of N—H⋯O hydrogen bonds.

## Related literature

For general background to imidazolidine derivatives, see: Tsao *et al.* (1991 ▶); Wang *et al.* (1995 ▶). For bond-length data, see: Allen *et al.* (1987 ▶). For hydrogen-bond motifs, see: Bernstein *et al.* (1995 ▶). For ring conformational analysis, see: Cremer & Pople (1975 ▶); Nardelli (1995 ▶).
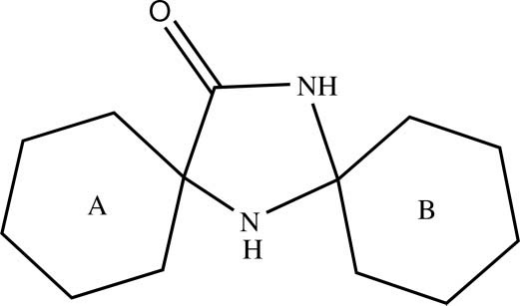


## Experimental

### 

#### Crystal data

C_13_H_22_N_2_O
*M* *_r_* = 222.33
Triclinic, 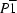

*a* = 5.8270 (8) Å
*b* = 10.1703 (5) Å
*c* = 10.6651 (4) Å
α = 86.103 (2)°
β = 81.331 (3)°
γ = 89.720 (3)°
*V* = 623.36 (9) Å^3^
*Z* = 2
Mo *K*α radiation
μ = 0.08 mm^−1^
*T* = 293 K
0.20 × 0.15 × 0.15 mm

#### Data collection

Bruker Kappa APEXII area-detector diffractometer
Absorption correction: multi-scan (*SADABS*; Sheldrick, 2001 ▶) *T*_min_ = 0.985, *T*_max_ = 0.989
11727 measured reflections
2311 independent reflections
2023 reflections with *I* > 2σ(*I*)
*R*_int_ = 0.020

#### Refinement

*R*[*F* ^2^ > 2σ(*F* ^2^)] = 0.039
*wR*(*F* ^2^) = 0.104
*S* = 1.04
2311 reflections
153 parameters
H atoms treated by a mixture of independent and constrained refinement
Δρ_max_ = 0.22 e Å^−3^
Δρ_min_ = −0.16 e Å^−3^

### 

Data collection: *APEX2* (Bruker, 2004 ▶); cell refinement: *SAINT* (Bruker, 2004 ▶); data reduction: *SAINT*; program(s) used to solve structure: *SHELXS97* (Sheldrick, 2008 ▶); program(s) used to refine structure: *SHELXL97* (Sheldrick, 2008 ▶); molecular graphics: *PLATON* (Spek, 2009 ▶); software used to prepare material for publication: *SHELXL97* and *PARST* (Nardelli, 1995 ▶).

## Supplementary Material

Crystal structure: contains datablocks I, global. DOI: 10.1107/S1600536810012468/ci5049sup1.cif

Structure factors: contains datablocks I. DOI: 10.1107/S1600536810012468/ci5049Isup2.hkl

Additional supplementary materials:  crystallographic information; 3D view; checkCIF report

## Figures and Tables

**Table 1:** Hydrogen-bond geometry (Å, °)

*D*—H⋯*A*	*D*—H	H⋯*A*	*D*⋯*A*	*D*—H⋯*A*
N3—H3⋯O1^i^	0.87 (2)	2.02 (2)	2.8821 (14)	172 (1)

Symmetry code: (i) 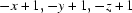.

**Fig. 1.**
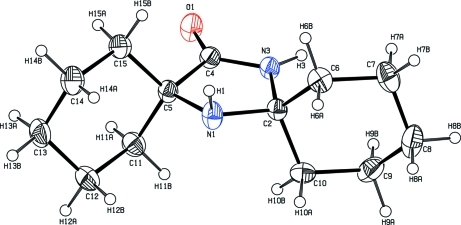
The molecular structure of the title compound. Displacement ellipsoids are drawn at the 30% probability level. H atoms are shown as circles with arbitrary radii.

**Fig. 2.**
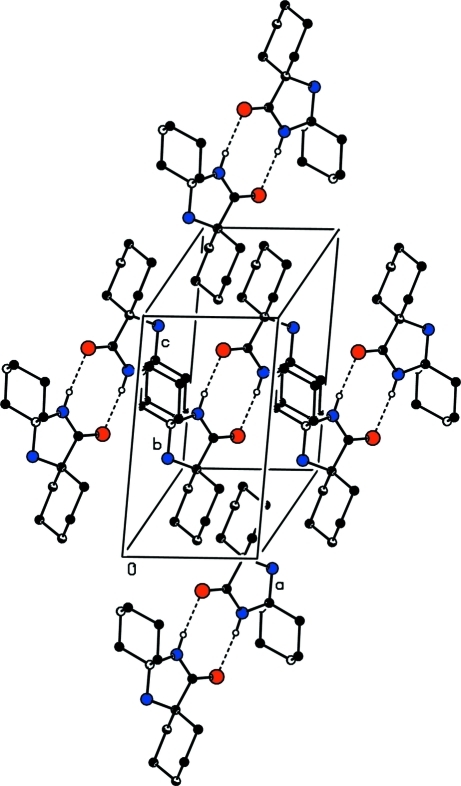
Crystal packing of the title compound. Dashed line indicate hydrogen bonds. H atoms not involved in hydrogen bonding (dashed lines) have been omitted for clarity.

## supplementary crystallographic information

### Comment

Imidazolidines occupy a unique position among the five-membered heterocycles and are highly used in synthetic as well as mechanistic organic chemistry and biochemistry (Tsao *et al.*, 1991). Imidazolidine derivatives are important intermediates and building blocks in the construction of various biologically active compounds (Wang *et al.*, 1995).

In the title molecule (Fig. 1), the five-membered imidazolidine ring is transfused with two cyclohexane rings. The bond lengths are comparable to the reported values (Allen *et al.*, 1987). The imidazolidine ring adopts an envelope conformation, with flap atom N1 deviating by 0.198 (2) Å from the C2/N3/C4/C5 plane. The asymmetry parameters for the imidazolidine ring shows that a mirror plane is passing through the atom N1 [ΔC_s_ = 2.7 (1)] (Nardelli, 1995); the puckering parameters [q2 = 0.128 (1) Å and φ(2) = 187.8 (5)°] (Cremer & Pople, 1975) also support the above fact. The sum of the bond angles around N1 (326.6°) shows sp^3^ hybridization and atom N3 (359.6°) is in accordance with sp^2^ hybridization. The two cyclohexane rings adopt chair conformations.

In the crystal, molecules are linked into centrosymmetric R_2_^2^(8) (Bernstein *et al.*, 1995) dimers by pairs of N—H···O hydrogen bonds (Table 1).

### Experimental

Potassium cyanide (20 mmol), ammonium chloride (20 mmol) and aqueous ammonium sulfide (30 ml) were dissolved in water (50 ml). Cyclohexanone (40 mmol) was slowly added into the above reaction mixture and stirred for 8 h at 333 K. The precipitated cyclohexan-1-spiro-2'-(imidazolidin-4'-thione)-5'-spiro-1''-cyclohexane was filtered. An ice-cold solution of the above imidazolidin-4-thione (5 mm0l) in glacial acetic acid (5 ml) was treated with hydrogen peroxide (30%, 5 ml) and kept at room temperature for 24 h. The reaction mixture was poured into crushed ice and extracted with ether (40 ml). Evaporation of ether yielded the title compound which was recrystallized by slow evaporation of a water–acetone (20:2) solution.

### Refinement

N-bound H atoms were located in a difference map and refined freely. C-bound H atoms were positioned geometrically (C—H = 0.97 Å) and allowed to ride on their parent atoms, with U_iso_(H) = 1.2U_eq_(C).

### Crystal data

**Table:**

C_13_H_22_N_2_O	*Z* = 2
*M**_r_* = 222.33	*F*(000) = 244
Triclinic, *P*1	*D*_x_ = 1.184 Mg m^−^^3^
Hall symbol: -P 1	Mo *K*α radiation, λ = 0.71073 Å
*a* = 5.8270 (8) Å	Cell parameters from 2023 reflections
*b* = 10.1703 (5) Å	θ = 2.7–25.5°
*c* = 10.6651 (4) Å	µ = 0.08 mm^−^^1^
α = 86.103 (2)°	*T* = 293 K
β = 81.331 (3)°	Block, colourless
γ = 89.720 (3)°	0.20 × 0.15 × 0.15 mm
*V* = 623.36 (9) Å^3^	

### Data collection

**Table:**

Bruker Kappa APEXII area-detector diffractometer	2311 independent reflections
Radiation source: fine-focus sealed tube	2023 reflections with *I* > 2σ(*I*)
graphite	*R*_int_ = 0.020
ω scans	θ_max_ = 25.5°, θ_min_ = 2.7°
Absorption correction: multi-scan (*SADABS*; Sheldrick, 2001)	*h* = −7→7
*T*_min_ = 0.985, *T*_max_ = 0.989	*k* = −12→12
11727 measured reflections	*l* = −12→12

### Refinement

**Table:**

Refinement on *F*^2^	Primary atom site location: structure-invariant direct methods
Least-squares matrix: full	Secondary atom site location: difference Fourier map
*R*[*F*^2^ > 2σ(*F*^2^)] = 0.039	Hydrogen site location: inferred from neighbouring sites
*wR*(*F*^2^) = 0.104	H atoms treated by a mixture of independent and constrained refinement
*S* = 1.04	*w* = 1/[σ^2^(*F*_o_^2^) + (0.0492*P*)^2^ + 0.1487*P*] where *P* = (*F*_o_^2^ + 2*F*_c_^2^)/3
2311 reflections	(Δ/σ)_max_ = 0.001
153 parameters	Δρ_max_ = 0.22 e Å^−^^3^
0 restraints	Δρ_min_ = −0.16 e Å^−^^3^

### Special details

**Table:**

Geometry. All esds (except the esd in the dihedral angle between two l.s. planes) are estimated using the full covariance matrix. The cell esds are taken into account individually in the estimation of esds in distances, angles and torsion angles; correlations between esds in cell parameters are only used when they are defined by crystal symmetry. An approximate (isotropic) treatment of cell esds is used for estimating esds involving l.s. planes.
Refinement. Refinement of *F*^2^ against ALL reflections. The weighted *R*-factor wR and goodness of fit *S* are based on *F*^2^, conventional *R*-factors *R* are based on *F*, with *F* set to zero for negative *F*^2^. The threshold expression of *F*^2^ > σ(*F*^2^) is used only for calculating *R*-factors(gt) etc. and is not relevant to the choice of reflections for refinement. *R*-factors based on *F*^2^ are statistically about twice as large as those based on *F*, and *R*- factors based on ALL data will be even larger.

### Fractional atomic coordinates and isotropic or equivalent isotropic displacement parameters (Å^2^)

**Table:**

	*x*	*y*	*z*	*U*_iso_*/*U*_eq_	
N1	0.1812 (2)	0.23573 (12)	0.32312 (10)	0.0440 (3)	
C2	0.17511 (19)	0.25258 (11)	0.45994 (11)	0.0320 (3)	
N3	0.32230 (18)	0.36886 (10)	0.46055 (9)	0.0348 (3)	
C4	0.4384 (2)	0.40747 (12)	0.34701 (11)	0.0348 (3)	
C5	0.3691 (2)	0.31781 (12)	0.24982 (11)	0.0346 (3)	
C6	−0.0713 (2)	0.27667 (14)	0.52388 (13)	0.0438 (3)	
H6A	−0.1707	0.2053	0.5079	0.053*	
H6B	−0.1280	0.3579	0.4866	0.053*	
C7	−0.0869 (3)	0.28619 (17)	0.66709 (14)	0.0553 (4)	
H7A	−0.0018	0.3634	0.6834	0.066*	
H7B	−0.2480	0.2964	0.7042	0.066*	
C8	0.0116 (3)	0.16453 (18)	0.72887 (14)	0.0630 (5)	
H8A	−0.0819	0.0882	0.7193	0.076*	
H8B	0.0065	0.1748	0.8190	0.076*	
C9	0.2598 (3)	0.14319 (15)	0.66826 (14)	0.0544 (4)	
H9A	0.3184	0.0629	0.7061	0.065*	
H9B	0.3557	0.2160	0.6845	0.065*	
C10	0.2759 (2)	0.13315 (12)	0.52573 (13)	0.0416 (3)	
H10A	0.1936	0.0547	0.5101	0.050*	
H10B	0.4375	0.1239	0.4892	0.050*	
C11	0.5811 (2)	0.23629 (14)	0.20042 (13)	0.0445 (3)	
H11A	0.7096	0.2953	0.1672	0.053*	
H11B	0.6265	0.1814	0.2704	0.053*	
C12	0.5332 (3)	0.14931 (15)	0.09662 (13)	0.0529 (4)	
H12A	0.6733	0.1022	0.0657	0.064*	
H12B	0.4145	0.0848	0.1315	0.064*	
C13	0.4534 (3)	0.23098 (16)	−0.01285 (13)	0.0577 (4)	
H13A	0.5781	0.2893	−0.0532	0.069*	
H13B	0.4159	0.1731	−0.0756	0.069*	
C14	0.2427 (3)	0.31170 (16)	0.03279 (13)	0.0572 (4)	
H14A	0.1125	0.2531	0.0636	0.069*	
H14B	0.2020	0.3675	−0.0379	0.069*	
C15	0.2867 (3)	0.39759 (14)	0.13896 (12)	0.0467 (3)	
H15A	0.4029	0.4638	0.1048	0.056*	
H15B	0.1446	0.4429	0.1697	0.056*	
O1	0.58158 (18)	0.49753 (9)	0.32312 (8)	0.0496 (3)	
H3	0.345 (3)	0.4032 (16)	0.5305 (15)	0.053 (4)*	
H1	0.043 (4)	0.265 (2)	0.301 (2)	0.097 (7)*	

### Atomic displacement parameters (Å^2^)

**Table:**

	*U*^11^	*U*^22^	*U*^33^	*U*^12^	*U*^13^	*U*^23^
N1	0.0481 (7)	0.0532 (7)	0.0324 (6)	−0.0184 (5)	−0.0079 (5)	−0.0091 (5)
C2	0.0332 (6)	0.0333 (6)	0.0310 (6)	−0.0062 (5)	−0.0078 (5)	−0.0063 (5)
N3	0.0434 (6)	0.0335 (5)	0.0290 (5)	−0.0089 (4)	−0.0078 (4)	−0.0070 (4)
C4	0.0404 (6)	0.0332 (6)	0.0323 (6)	−0.0054 (5)	−0.0089 (5)	−0.0047 (5)
C5	0.0399 (6)	0.0357 (6)	0.0294 (6)	−0.0071 (5)	−0.0076 (5)	−0.0054 (5)
C6	0.0334 (7)	0.0506 (8)	0.0486 (8)	−0.0011 (6)	−0.0088 (5)	−0.0054 (6)
C7	0.0424 (8)	0.0719 (10)	0.0494 (8)	−0.0067 (7)	0.0067 (6)	−0.0177 (7)
C8	0.0770 (11)	0.0735 (11)	0.0361 (8)	−0.0247 (9)	−0.0025 (7)	0.0021 (7)
C9	0.0700 (10)	0.0470 (8)	0.0491 (8)	−0.0036 (7)	−0.0236 (7)	0.0091 (6)
C10	0.0433 (7)	0.0316 (6)	0.0512 (8)	−0.0003 (5)	−0.0098 (6)	−0.0051 (5)
C11	0.0459 (7)	0.0468 (7)	0.0424 (7)	0.0003 (6)	−0.0091 (6)	−0.0098 (6)
C12	0.0659 (9)	0.0470 (8)	0.0465 (8)	0.0030 (7)	−0.0041 (7)	−0.0166 (6)
C13	0.0811 (11)	0.0589 (9)	0.0338 (7)	−0.0059 (8)	−0.0051 (7)	−0.0151 (6)
C14	0.0735 (10)	0.0669 (10)	0.0363 (7)	0.0019 (8)	−0.0219 (7)	−0.0100 (7)
C15	0.0609 (9)	0.0459 (8)	0.0359 (7)	0.0046 (6)	−0.0135 (6)	−0.0063 (6)
O1	0.0645 (6)	0.0472 (5)	0.0371 (5)	−0.0263 (5)	−0.0055 (4)	−0.0052 (4)

### Geometric parameters (Å, °)

**Table:**

N1—C5	1.4724 (16)	C8—H8B	0.97
N1—C2	1.4759 (15)	C9—C10	1.5192 (19)
N1—H1	0.91 (2)	C9—H9A	0.97
C2—N3	1.4652 (14)	C9—H9B	0.97
C2—C6	1.5203 (17)	C10—H10A	0.97
C2—C10	1.5213 (17)	C10—H10B	0.97
N3—C4	1.3300 (16)	C11—C12	1.5213 (18)
N3—H3	0.872 (17)	C11—H11A	0.97
C4—O1	1.2296 (15)	C11—H11B	0.97
C4—C5	1.5255 (15)	C12—C13	1.516 (2)
C5—C15	1.5243 (18)	C12—H12A	0.97
C5—C11	1.5315 (18)	C12—H12B	0.97
C6—C7	1.5260 (19)	C13—C14	1.511 (2)
C6—H6A	0.97	C13—H13A	0.97
C6—H6B	0.97	C13—H13B	0.97
C7—C8	1.514 (2)	C14—C15	1.5281 (18)
C7—H7A	0.97	C14—H14A	0.97
C7—H7B	0.97	C14—H14B	0.97
C8—C9	1.514 (2)	C15—H15A	0.97
C8—H8A	0.97	C15—H15B	0.97
			
C5—N1—C2	109.28 (9)	C10—C9—H9A	109.4
C5—N1—H1	108.7 (14)	C8—C9—H9B	109.4
C2—N1—H1	107.6 (14)	C10—C9—H9B	109.4
N3—C2—N1	103.04 (9)	H9A—C9—H9B	108.0
N3—C2—C6	111.13 (10)	C9—C10—C2	112.71 (11)
N1—C2—C6	110.88 (10)	C9—C10—H10A	109.0
N3—C2—C10	110.55 (9)	C2—C10—H10A	109.0
N1—C2—C10	111.11 (10)	C9—C10—H10B	109.0
C6—C2—C10	109.97 (10)	C2—C10—H10B	109.0
C4—N3—C2	113.89 (9)	H10A—C10—H10B	107.8
C4—N3—H3	123.1 (10)	C12—C11—C5	112.13 (11)
C2—N3—H3	122.6 (10)	C12—C11—H11A	109.2
O1—C4—N3	126.68 (11)	C5—C11—H11A	109.2
O1—C4—C5	125.05 (11)	C12—C11—H11B	109.2
N3—C4—C5	108.25 (10)	C5—C11—H11B	109.2
N1—C5—C15	111.71 (11)	H11A—C11—H11B	107.9
N1—C5—C4	103.74 (9)	C13—C12—C11	110.91 (12)
C15—C5—C4	111.26 (10)	C13—C12—H12A	109.5
N1—C5—C11	112.27 (11)	C11—C12—H12A	109.5
C15—C5—C11	109.53 (10)	C13—C12—H12B	109.5
C4—C5—C11	108.18 (10)	C11—C12—H12B	109.5
C2—C6—C7	112.42 (11)	H12A—C12—H12B	108.0
C2—C6—H6A	109.1	C14—C13—C12	111.04 (12)
C7—C6—H6A	109.1	C14—C13—H13A	109.4
C2—C6—H6B	109.1	C12—C13—H13A	109.4
C7—C6—H6B	109.1	C14—C13—H13B	109.4
H6A—C6—H6B	107.9	C12—C13—H13B	109.4
C8—C7—C6	111.17 (12)	H13A—C13—H13B	108.0
C8—C7—H7A	109.4	C13—C14—C15	111.62 (12)
C6—C7—H7A	109.4	C13—C14—H14A	109.3
C8—C7—H7B	109.4	C15—C14—H14A	109.3
C6—C7—H7B	109.4	C13—C14—H14B	109.3
H7A—C7—H7B	108.0	C15—C14—H14B	109.3
C7—C8—C9	110.33 (12)	H14A—C14—H14B	108.0
C7—C8—H8A	109.6	C5—C15—C14	112.45 (11)
C9—C8—H8A	109.6	C5—C15—H15A	109.1
C7—C8—H8B	109.6	C14—C15—H15A	109.1
C9—C8—H8B	109.6	C5—C15—H15B	109.1
H8A—C8—H8B	108.1	C14—C15—H15B	109.1
C8—C9—C10	111.04 (12)	H15A—C15—H15B	107.8
C8—C9—H9A	109.4		
			
C5—N1—C2—N3	13.53 (13)	C10—C2—C6—C7	53.21 (14)
C5—N1—C2—C6	132.50 (11)	C2—C6—C7—C8	−55.77 (16)
C5—N1—C2—C10	−104.88 (12)	C6—C7—C8—C9	56.55 (17)
N1—C2—N3—C4	−9.88 (14)	C7—C8—C9—C10	−56.59 (17)
C6—C2—N3—C4	−128.68 (11)	C8—C9—C10—C2	55.99 (16)
C10—C2—N3—C4	108.91 (12)	N3—C2—C10—C9	69.59 (14)
C2—N3—C4—O1	−176.02 (12)	N1—C2—C10—C9	−176.64 (11)
C2—N3—C4—C5	2.38 (14)	C6—C2—C10—C9	−53.49 (14)
C2—N1—C5—C15	−132.29 (11)	N1—C5—C11—C12	69.59 (14)
C2—N1—C5—C4	−12.35 (13)	C15—C5—C11—C12	−55.11 (15)
C2—N1—C5—C11	104.22 (12)	C4—C5—C11—C12	−176.55 (11)
O1—C4—C5—N1	−175.37 (12)	C5—C11—C12—C13	56.86 (16)
N3—C4—C5—N1	6.20 (13)	C11—C12—C13—C14	−56.04 (17)
O1—C4—C5—C15	−55.12 (17)	C12—C13—C14—C15	54.99 (18)
N3—C4—C5—C15	126.44 (12)	N1—C5—C15—C14	−71.20 (15)
O1—C4—C5—C11	65.24 (16)	C4—C5—C15—C14	173.39 (12)
N3—C4—C5—C11	−113.19 (12)	C11—C5—C15—C14	53.83 (15)
N3—C2—C6—C7	−69.54 (14)	C13—C14—C15—C5	−54.77 (17)
N1—C2—C6—C7	176.48 (11)		

### Hydrogen-bond geometry (Å, °)

**Table:**

*D*—H···*A*	*D*—H	H···*A*	*D*···*A*	*D*—H···*A*
N3—H3···O1^i^	0.87 (2)	2.02 (2)	2.8821 (14)	172 (1)

Symmetry codes: (i) −*x*+1, −*y*+1, −*z*+1.
